# A letter from the new editor‐in‐chief

**DOI:** 10.1002/brb3.2967

**Published:** 2023-03-15

**Authors:** Amanda Bischoff‐Grethe

**Affiliations:** ^1^ Department of Psychiatry University of California San Diego La Jolla California USA

I am very honored to have been selected as the new editor‐in‐chief for *Brain and Behavior*.

*Brain and Behavior* was created in 2011 as an open‐access, multidisciplinary journal, poised to take advantage of the wide reach of the Internet for rapid publication (Alexandrov, 2011). At its heart, *Brain and Behavior* has been focused on the dissemination of good, sound science. A study does not need to be novel to be relevant. While novel findings make splashy headlines, it is the careful, confirmatory work that does them justice by demonstrating that the effect can be repeated in independent study samples. This is crucial to establish novel findings as replicable, as it can bring about a paradigm shift in the field or encourage new directions of research. *Brain and Behavior*’s philosophy is that good papers deserve to be seen, peer‐reviewed, read, and cited. Our commitment to sound science principles allows authors at every stage of their career to find a home for their paper with our journal, and we look forward to receiving your submissions.

I joined the editorial team in 2017 under the stewardship of Dr. Maryann Martone. Dr. Martone has long held an interest in open science and resource sharing, and she has been a champion of scientific rigor and transparency. During her tenure with *Brain and Behavior*, she was instrumental in encouraging authors to make data available (Martone, 2014). One approach she helped promote was the use of data articles, which allow researchers to describe the data they plan to deposit into an open‐source repository. Dr. Martone also helped establish the use of Research Resource Identifiers in publications to make the identification of resources more transparent. These and other efforts both support reproducibility as well as provide ways for authors and developers to promote their work to the broader community.

Dr. Nutan Sharma succeeded Dr. Martone in 2018. Over the past 5 years, Dr. Sharma oversaw a steady increase in manuscript submissions that substantially accelerated in 2020 and 2021 during the initial years of the Coronavirus Disease 2019 (COVID‐19) pandemic. As with other journals, this unprecedented increase posed a challenge to our editorial board, as there was no in‐kind increase in editorial staff, and members were dealing with their own unique challenges as we navigated the early stages of the pandemic. Yet, our board rose to the occasion, and we oversaw a 60% increase in the number of published articles. As an associate editor, it was a privilege to support Dr. Sharma, and I wish her every success in her future endeavors.

As editor‐in‐chief, it is my responsibility to build upon the advances of my predecessors and to ensure that *Brain and Behavior* continues to grow as a valued resource in the open science community. One of our plans is to promote special issues on topics well aligned with our journal's scope. These are excellent opportunities to highlight areas of research that are rapidly evolving or are underexplored in the literature. We would also like to welcome contributions by early career researchers and provide opportunities for junior guest editor roles that would enhance their professional development and help grow their networks. Second, while the submission rate has stabilized, we are still experiencing significant challenges in the review process due to the overwhelming number of submissions relative to the size of our editorial board. Over the coming months, we plan to add new members to our editorial team and expand the editorial board, which will help to process manuscript submissions more efficiently and provide a smooth author experience. Finally, we will continue our support of ethics in publishing and explore new ways to combat fraudulent research. There is often the misperception that open‐access journals engage in less stringent reviews. This is untrue, as we engage in the same rigorous standards as hybrid and subscription journals to ensure manuscripts adhere to good science, with a clear description of the methods and a thoughtful discussion of the study outcome. Our dedicated ethics team meets regularly to identify and prevent potential threats to the scientific literature.

I look forward to my evolving role and continued work to support *Brain and Behavior*’s goals to promote sound science.

### PEER REVIEW

The peer review history for this article is available at https://publons.com/publon/10.1002/brb3.2967.
